# Identification of cancer-associated missense mutations in *hace1* that impair cell growth control and Rac1 ubiquitylation

**DOI:** 10.1038/srep44779

**Published:** 2017-03-20

**Authors:** Emilie Andrio, Romain Lotte, Daniel Hamaoui, Jacqueline Cherfils, Anne Doye, Mads Daugaard, Poul H. Sorensen, Frédéric Bost, Raymond Ruimy, Amel Mettouchi, Emmanuel Lemichez

**Affiliations:** 1 Inserm, U1065, C3M, Team Bacterial Toxins in Host-Pathogens Interactions France, Equipe labellisée la ligue contre le cancer, Nice 06204, France; 2 Université Nice Côte d’Azur, Inserm, C3M, France; 3 Laboratoire de Bactériologie, CHU de Nice, Hôpital l′Archet, Nice, France; 4 Laboratoire de Biologie et Pharmacologie Appliquée, CNRS et Ecole Normale Supérieure Paris-Saclay, Université Paris-Saclay, Cachan, France; 5 Vancouver Prostate Center, Vancouver, British Columbia, Canada; 6 Department of Urologic Sciences, University of British Columbia, Vancouver, British Columbia, Canada; 7 Department of Molecular Oncology, British Columbia Cancer Research Center, Vancouver, Canada; 8 Inserm U1065, C3M, Team Cellular and Molecular Physiopathology of Obesity and Diabetes, Nice 06204, France

## Abstract

The E3 ubiquitin ligase HACE1 is a potent tumor suppressor that controls cell proliferation and ubiquitylates the small GTPase Rac1 to target it to proteasomal degradation. Whether and how the activity of HACE1 is regulated by the N-terminal ankyrin (ANK) and the middle (MID) domains is ill defined. Here, we identified in the version 64 of the Catalogue of Somatic Mutations in Cancer (COSMIC) 13 missense mutations of *hace1* located outside the HECT domain, and found that all lead to defective control of cell proliferation. In addition, several mutations located in the ankyrin domain displayed a dramatic reduction in Rac1 ubiquitylation associated with a decrease of colony formation in soft agar. 3D structure modelling of the 7 ankyrin-repeats coupled to functional analysis identified a surface epitope centered on one of the mutated residue, Gly-175, which is critical for controlling Rac1 binding and ubiquitylation. We also identified a role for the MID domain in conferring the specificity of association of HACE1 to the active form of Rac1. Our study of the functional interplay between HACE1 and Rac1 in cancer thus sheds a new light on the molecular mechanism of Rac1 ubiquitylation by HACE1 and the impact of its cancer-associated mutations in cell proliferation.

The abnormal expression of mammalian HECT family E3 ubiquitin-protein ligases is involved in age-related diseases notably cancer but little is known about missense mutations in these proteins and how they affect their function^1^,^2^,^3^. With the development of personalized medicine, which involves systematic exome sequencing of tumors, we can now identify cancer-associated missense mutations to study their effect.

E3 ubiquitin ligases are involved in a broad range of cellular homeostatic processes including cell signaling, clearance of misfolded proteins, cell cycle progression and apoptosis. They catalyze the addition of one or several ubiquitin polypeptides to target proteins. HECT-domain and Ankyrin-repeat Containing E3 ubiquitin protein ligase 1 (HACE1) is an E3 ubiquitin ligase with an important role in cancer that was first unveiled by drawing a genetic link between *hace1* loss of expression and sporadic Wilms’ tumors^4^. This was followed by the report that mice knockout for *hace1* developed spontaneously cancers originating from the three germ layers during aging, pointing to a protective function of HACE1 in cell homeostasis^5^. Following these first demonstrations of HACE1 tumor suppressor role, loss of expression of *hace1* upon epigenetic silencing or chromosomal translocations has been associated with the development of multiple types of human cancers^5^,^6^,^7^,^8^,^9^. More recently, loss of *hace1* expression was shown to be crucial for HER2/neu-dependent breast cancer progression^10^. The loss of HACE1 expression that has been reported in a broad spectrum of human tumors suggests that HACE1 functions could also be affected by missense mutations, but this has remained an unexplored issue. Here we addressed this question by taking advantage of tumor exome sequencing programs.

The small GTPase Rac1 is a well-established target of the ubiquitin ligase activity of HACE1^10^,^11^,^12^,^13^. This E3 ubiquitin ligase catalyzes the ubiquitylation of Rac1 once it is activated by point-mutations (Q61L, Q61E or G12V), expression of the GEF-domain of Dbl or in response to the activation of c-Met and HER2/neu signaling pathways^10^,^11^,^12^. The poly-ubiquitylation of Rac1 catalyzed by HACE1 targets this GTPase to the proteasomal degradation machinery^10^,^11^,^12^. The cellular level of Rac1 increases in *hace1* KO mice and Wilms’ tumors deficient in HACE1^13^. The re-expression of HACE1 in MEF cells isolated from *hace1* KO mice restores normal Rac1 levels^12^. HACE1-induced degradation of Rac1 controls the amplitude and duration of Rac1 activity with critical consequences for cell migration and proliferation^10^,^12^,^13^. In link with its capacity to hamper Rac1 signaling, HACE1 bears a critical guard function against oxidative stress. Its loss of expression leads to accumulation of reactive oxygen species, responsible for neurodegeneration, DNA damages, deregulation of cyclin D1-mediated control of the cell cycle progression and metabolic reprograming of cells, events involved in cell transformation^13^,^14^,^15^.

HACE1 is characterized by the presence of an amino-terminal domain containing several ankyrin repeats (ANK domain) located upstream a middle region (MID domain, residues 258–576) and the catalytic HECT domain (Fig. 1a). Cysteine 876 in the HECT domain is critical for the catalysis of ubiquitin transfer from an E2 enzyme to a lysine residue of target proteins^4^. The MID domain has no homology to any domain of known function or structure. Ankyrin repeats classically form a protein-protein interaction motif with a well conserved architecture, which is found in a large set of proteins with essential functions^16^. Deletion of the ANK domain impairs the capacity of HACE1 to interact with Rac1^12^. However, the role of the ANK and MID domains in the control of Rac1 ubiquitylation has remained poorly characterized.

Here, we reasoned that cancer-associated mutations in *hace1* might reveal the molecular and functional relationship between the tumor-suppressive function of HACE1 and the cell growth-promoting activity of Rac1. We identify for the first time cancer-associated missense mutations that alter HACE1 regulatory function in cell proliferation. Moreover, we identify in HACE1 a cluster of surface exposed amino-acids in ankyrin repeats 5 and 6, which controls its binding to, and ubiquitylation of Rac1, as well as a role for the MID domain in conferring the specificity of association to the active form of Rac1.

## Results

### Cancer-associated missense mutations outside the HECT domain abolish HACE1 control of cell growth

**Figure 1:**
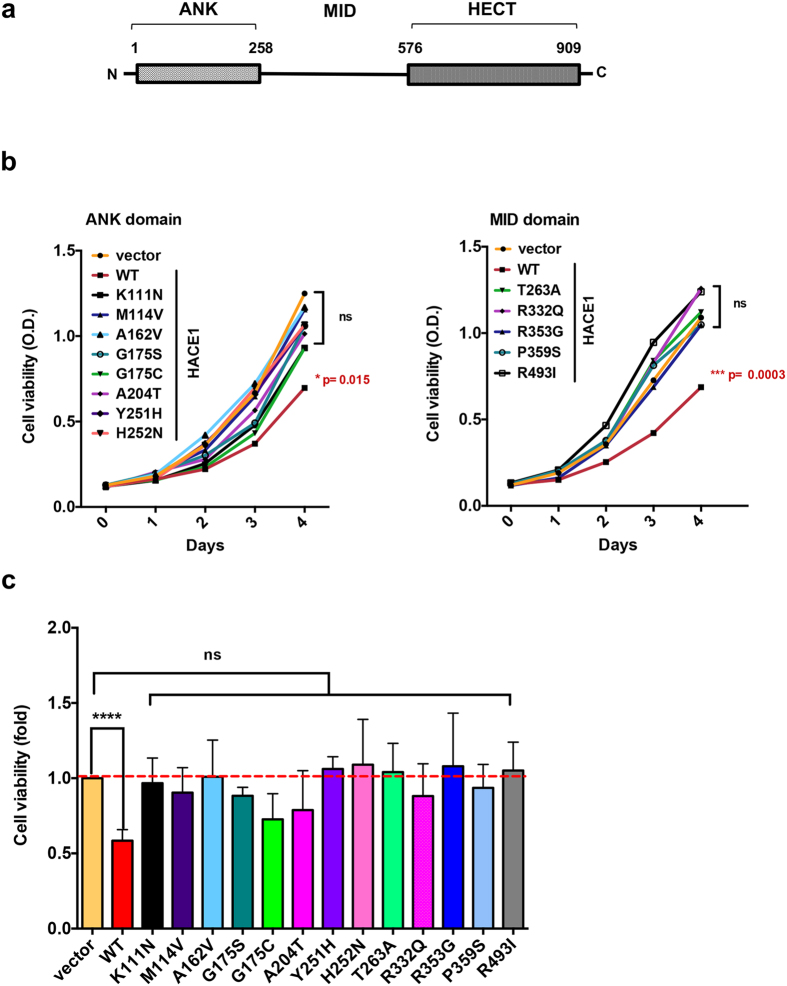
Ability of HACE1 mutants to control cell proliferation. (**a**) Schematic representation of the three domains of HACE1 encompassing the ankyrin-repeats (ANK 1–258), middle (MID 258–576) and HECT (576–909) domains with indicated amino acid positions. (**b**) Graphs show kinetics of cell proliferation. MCF-12A cell lines expressing HACE1 wild-type or mutants either located in the ANK (left panel) or in the MID (right panel) domain. Each value corresponds to mean values of three independent experiments with technical quadruplicate analyzed by one-way ANOVA Dunnett’s multiple comparison test with ***p ≤ 0.0003, *p ≤ 0.015 or non-significant (ns). (**c**) Graph shows quantification of cell growth 4 days after cell seeding of HA-HACE1 wild-type (WT) and mutants expressing cells relative to mock transduced cells. Measurements were performed four days after cell seeding by XTT analysis. Data correspond to mean ± SEM (n ≥ 3 biological replicates each in quadruplicates). Data analyzed by one-way ANOVA Dunnett’s multiple comparison test with ****p < 0.0001 or non-significant (ns).

To gain insight into the function of the non-catalytic ANK and MID domains of HACE1 and their role in cancer, we reasoned that missense mutations identified in cancer samples in these domains may reflect defects in their functions leading to impaired control of cell proliferation. Our search of the Catalogue of Somatic Mutations in Cancer (COSMIC, version 64) database identified 13 missense mutations located in the ANK-MID region of HACE1 (amino-acids 1–545; Supplementary Table 1). We examined the impact of expression of each individual mutated form of HACE1 on cell growth. Each of the 13 missense mutations was introduced into the HA-tagged sequence of wild-type *hace1* cloned in a retroviral vector. Loss of HACE1 expression in MCF-12A mammary epithelial cells leads to enhanced Rac1 activity that is essential for cell proliferation^10^. We thus transduced this cell line to generate stable cell lines expressing each of the mutated allele of *hace1* (Supplementary Figure S1a). We next established the kinetics of growth of cells stably expressing HACE1 wild-type and mutants compared to mock transduced cells, as a control. Growth kinetics are presented in Fig. 1b. Interestingly, the restriction of proliferation conferred by expression of wild-type HACE1 was diminished by all the cancer-associated mutations identified in our study. Each mutation had similar impact on the control of cell proliferation by HACE1 (Fig. 1c).

We concluded from this analysis that missense mutations found in cancer samples outside the catalytic domain of HACE1 alleviate its capacity to bridle cell proliferation.

### Characterization of HACE1 mutants with impaired E3 ligase activity on Rac1

**Figure 2:**
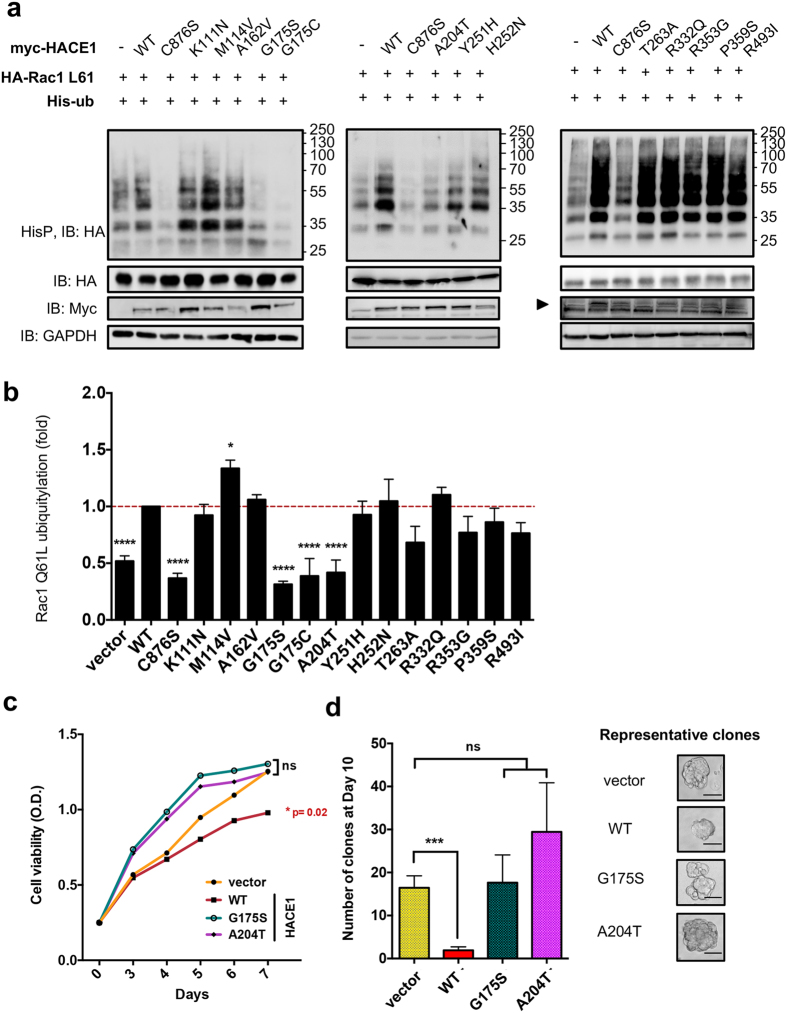
Efficiency of Rac1 ubiquitylation by cancer-associated mutants of HACE1. (**a**) Immunoblots showing levels of Rac1 ubiquitylation catalyzed by HACE1 wild-type (WT), catalytically inactive C876S mutant and 13 identified cancer-associated missense mutations indicated in the panels. Precipitated His_6_-tagged ubiquitylated proteins (HisP) were immunoblotted anti-HA to reveal the ubiquitylation level of Rac1 Q61L. Immunoblots anti-HA and anti-myc show control expression of HA-tagged Rac1L61 and myc-tagged HACE1 WT and mutants (arrowhead). Immunoblot anti-GAPDH is used as loading control. (**b**) Graph showing the quantification of Rac1 Q61L ubiquitylation in cells expressing the different HACE1 mutants. Values are expressed as fold variations compared to HACE1 WT condition. Data correspond to mean ± SEM (n ≥ 4 biological replicates). Data were analyzed by one-way ANOVA Dunnett’s multiple comparison test with *p < 0.05, ****p < 0.0001 or non-significant (ns). (**c**) Graph show kinetics of proliferation of MCF7 cells stably expressing HACE1 Wild-type, G175S or A204T mutants. Data correspond to mean ± SEM (n = 3 independent experiments performed in technical quadruplicate) analyzed by one-way ANOVA Dunnett’s multiple comparison test: *p < 0.02 or non-significant (ns). (**d**) Anchorage-independent cell growth of MCF7 expressing HACE1 wild-type, G175S or A204T mutants. Examples of clones counted are shown. Scale bar = 100 μm. Data correspond to mean ± SEM (n = 3 independent experiments performed in technical quadruplicate) analyzed by unpaired student t-test (***p < 0.0009 or non-significant, ns).

Considering that Rac1 signaling promotes cell proliferation, we searched for a possible interplay between the mutations of HACE1 and its ubiquitin-ligase activity on this GTPase. To this aim, we conducted an ubiquitylation-based screening approach. We compared the efficiency of ubiquitylation of wild-type or mutated forms of HACE1 on Rac1 Q61L (Fig. 2a and b). As previously reported, we found that wild-type HACE1 expression increased Rac1 ubiquitylation levels. Conversely, expression of the catalytically inactive HACE1 C876S mutant resulted in a dominant negative effect on Rac1 ubiquitylation. We used these two constructs to define the maximum and minimum reference levels of ubiquitylation of Rac1, respectively. None of the mutations introduced in the MID domain, i.e. T263A, R332Q, R353G, P359S and R493I, had a significant impact on Rac1 ubiquitylation. In contrast, we identified four mutations in the ANK domain that affected Rac1 ubiquitylation (Fig. 2a and b). Remarkably, three of these mutations, G175S, G175C and A204T, displayed a reduced efficiency of Rac1 ubiquitylation similar to that of the catalytic inactive mutant C876S (Fig. 2b). Although we failed to detect the active form of endogenous Rac1 by GST-Pak pulldown, we found that mutations G175S, G175C and A204T abrogate the capacity of HACE1 to reduce total Rac1 level in MCF-12A cells (Supplementary Figure S1b). Having established the critical importance of amino acids in position 175 and 204 in the control of Rac1 ubiquitylation, we assessed the impact of cancer-associated mutations at these positions on anchorage-independent cell growth. At first, we transduced human mammary adenocarcinoma MCF7 transformed cells with *hace1* wild-type and mutants and measured growth kinetics. Expression of HACE1 mutants, opposite to the wild-type form, had no impact on cell proliferation (Fig. 2c and Supplementary Figure S1c). Moreover, when we assessed soft agar colony formation, we found that only wild-type HACE1 reduced the number and size of the clones (Fig. 2d).

Altogether, this analysis of cancer-associated missense mutations in *hace1* allowed us to identify two major residues Gly-175 and Ala-204 in the ANK domain that are critical for both Rac1 ubiquitylation and cell growth restriction.

### Structure-function analysis of HACE1 mutants

**Figure 3:**
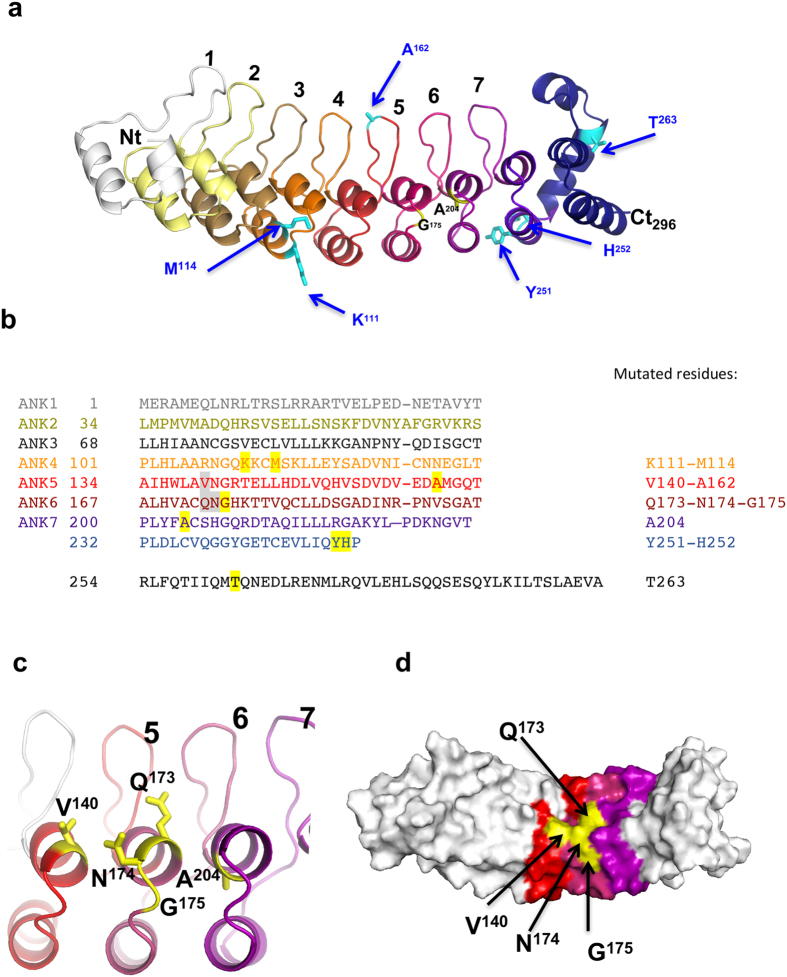
HACE1 mutant localization on the 3D model of HACE1 ankyrin repeats. (**a**) Structural model of HACE1 calculated with the Robetta server. The model depicts the 7 ankyrin repeats at HACE1 N-terminus (residues 1–234, color-coded and labelled) followed by an incomplete ankyrin repeat (residues 235–253, dark violet) and an α-helical region (residues 254–296, dark blue). Cancer-associated missense mutations are reported on the structure. Mutation of residues in black reduced Rac1 ubiquitylation. Mutants depicted in cyan show no or slight increase in Rac1 ubiquitylation. (**b**) Primary sequence of HACE1 ankyrin repeats (amino acids 1–296). The 7 repeats and the incomplete 8^th^ repeat are aligned based on the structural model. Residues 254–263 are predicted to form a helical structure that caps the last ankyrin repeat. Residues described in the text are highlighted in grey (functional epitope) and yellow (COSMIC mutations). The corresponding residues are given on the right. (**c**) Close-up view centered on HACE1 ankyrin repeats 5, 6 and 7 depicting surface-located amino acids V140, Q173, N174, G175. The alanine in position 204 is located on the internal part of the helix. The orientation is identical as in (**a**). (**d**) Representation of HACE1 ankyrin repeats depicting the cluster of residues controlling Rac1 ubiquitylation (yellow) at the surface of the repeats 5, 6 and 7.

**Figure 4:**
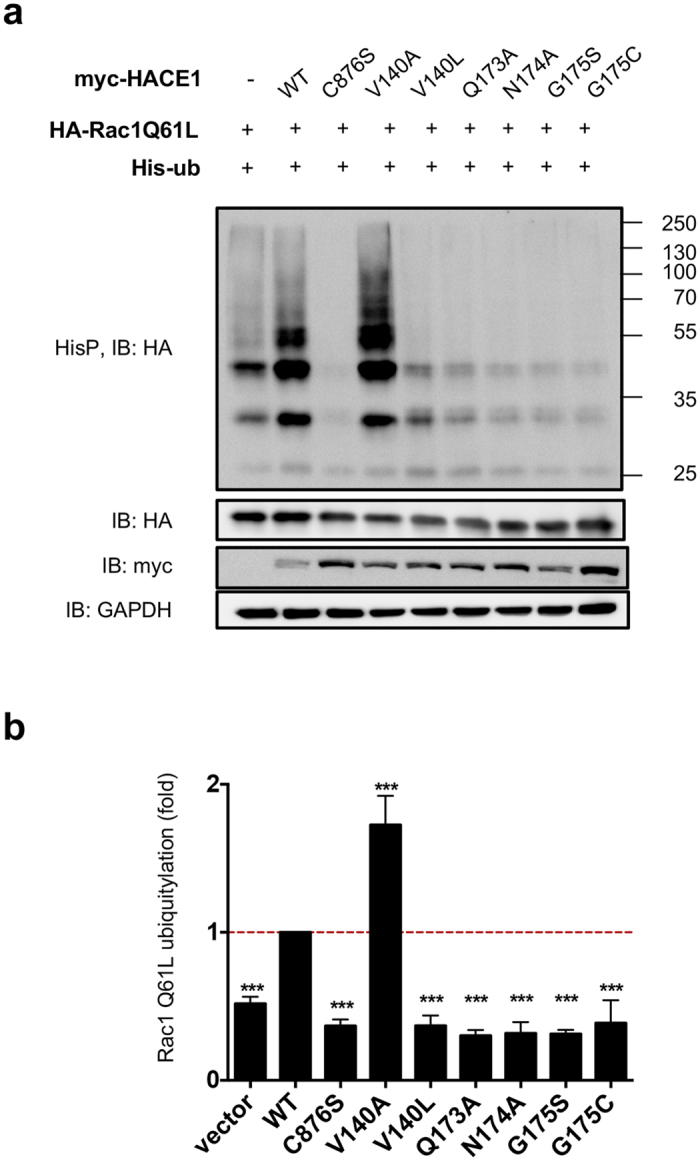
Mutations in HACE1 that modulate its ubiquitylation activity on Rac1. (**a**) Immunoblots showing levels of ubiquitylation of Rac1 Q61L by HACE1 wild-type (WT), catalytically inactive C876S mutant and mutants of HACE1 indicated in the panel. Precipitated His_6_-tagged ubiquitylated proteins (HisP) were immunoblotted (IB) anti-HA to reveal Rac1 Q61L ubiquitylation profile. Immunoblots anti-HA and anti-myc on total cell lysates show protein expression and anti-GAPDH is used as loading control. (**b**) Graph shows quantification of the ubiquitylation of Rac1 Q61L by HACE1 wild-type (WT) and mutants. Values are expressed as fold variations compared to HACE1 WT condition, that was set to a value of 1. Data correspond to mean ± SEM (n ≥ 4 biological replicates). Data were analyzed by one-way ANOVA Dunnett’s multiple comparison test with ***p < 0.001.

The above data pointed toward a role of the N-terminal ANK domain of HACE1 in the control of Rac1 ubiquitylation. Ankyrin repeats are known to pack into regular structures that can reliably be modeled by protein structure prediction algorithms^16^. A model structure of the amino-terminal third part of HACE1, encompassing residues 1–296, was calculated with the Robetta server, showing unambiguously the presence of 7 ankyrin repeats, followed by a helical structure that caps an incomplete 8^th^ ankyrin repeat (Fig. 3a). This model allowed us to locate the positions of COSMIC mutants on ankyrin repeats (Fig. 3a and b). Mutations affecting Rac1 ubiquitylation at amino acids Gly-175 and Ala-204, face each other on repeats 6 and 7 (Fig. 3c). Comparison of the sequence of HACE1 with other ANK domain-containing proteins showed that the amino acid at position 175 is highly conserved and is either a glycine or asparagine residue. Both these residues are thought to introduce a tight turn between the two helices in the ankyrin repeat motif. Therefore, we tested the effect of the G175N substitution, which should modify the exposed side chain without affecting the folding of the repeats. We measured that this mutant had an inhibitory effect on the ubiquitylation of Rac1 (Supplementary Figure S2). Thus, despite the fact that both amino acids can conservatively be found at this position, suggesting that the ankyrin motif folding is correct, they are not functionally equivalent in term of activities. To further investigate whether these residues identify a functional epitope, we designed additional mutations at position Gln-173, Asn-174 and Val-140, which are located at the surface of the domain in the vicinity of Gly-175 (Fig. 3c and d). We observed that all these mutations had a strong impact on the efficiency of ubiquitylation of Rac1 (Fig. 4a and b). We found that the substitution of Val-140 into an alanine residue markedly increased the efficiency of ubiquitylation of Rac1, whereas the substitution of Val-140 into a bulkier leucine residue decreased Rac1 ubiquitylation (Fig. 4a and b). This suggests that the size of the side chain at position 140 determines the impact of the mutation on Rac1 ubiquitylation. Assessing the biological effect of V140A revealed that it kept the capacity to control cell growth (Supplementary Figure S3). Mutations at position 173 and 174 diminished HACE1 ubiquitylation activity on Rac1, similar to mutations G175C, G175S and V140L (Fig. 4a and b). Altogether, we identified a cluster of four critical amino-acids at the surface of Ankyrin repeats that controls Rac1 ubiquitylation.

Collectively, we identified a functional site in HACE1 ankyrin repeats 4 to 7 that controls the efficiency of Rac1 ubiquitylation.

### Cancer-associated somatic mutations of HACE1 modulate Rac1 binding

**Figure 5:**
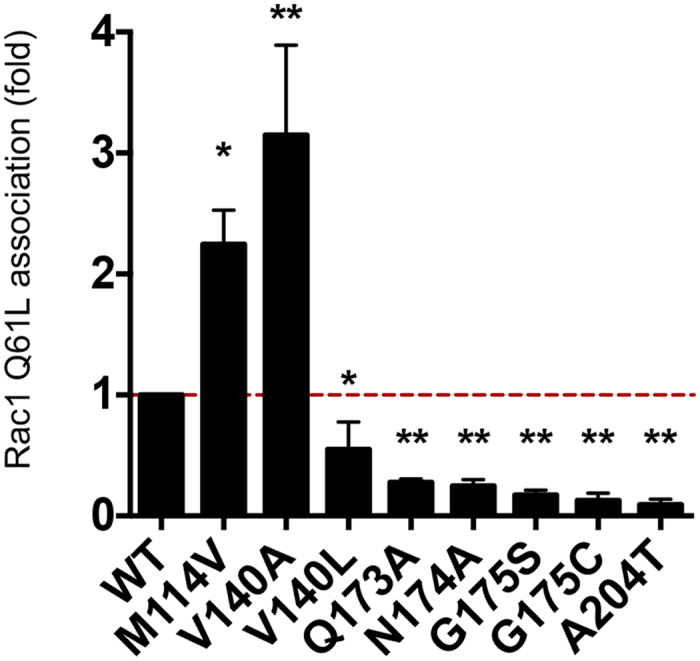
Mutations in HACE1 that modulate its binding to Rac1. Quantification of anti-myc co-immunoprecipitations (IP) from cells co-transfected with expression vectors for myc-Rac1 Q61L and the HA-tagged mutants of HACE1. Values are expressed as fold variations compared to condition of wild-type HACE1 co- immunoprecipitation with myc-Rac1 Q61L set to the value of 1. Data correspond to mean ± SEM, (n ≥ 4 biological replicates). Data were analyzed by one-way ANOVA Dunnett’s multiple comparison test with *p < 0.05; **p < 0.001.

We then evaluated the binding of active Rac1 to HACE1 mutants. We compared the capacity of HACE1 wild-type and mutants to interact with the active form of Rac1 by co-immunoprecipitations using the constitutively active Rac1 Q61L mutant as a bait. Mutants V140L, Q173A, N174A, G175S, G175C, G175N and A204T, which were impaired for Rac1 ubiquitylation, had a dramatic decrease in their interaction with Rac1 Q61L (Fig. 5 and Supplementary Figure S4). Conversely, mutants V140A and M114V to a lesser extent, showed a gain of interaction in good agreement with their increase in ubiquitylation activity on Rac1 (Fig. 5 and Supplementary Figure S4). Altogether, these data identify critical amino-acids functional site in HACE1 ankyrin repeats 4 to 7 that control the interaction of HACE1 with Rac1, thereby establishing a correlation between the efficiency of interaction of Rac1 with HACE1 and the efficiency of ubiquitylation of Rac1 (R^2^ = 0,87).

### The ANK and MID domains cooperate to bind selectively to active Rac1

**Figure 6:**
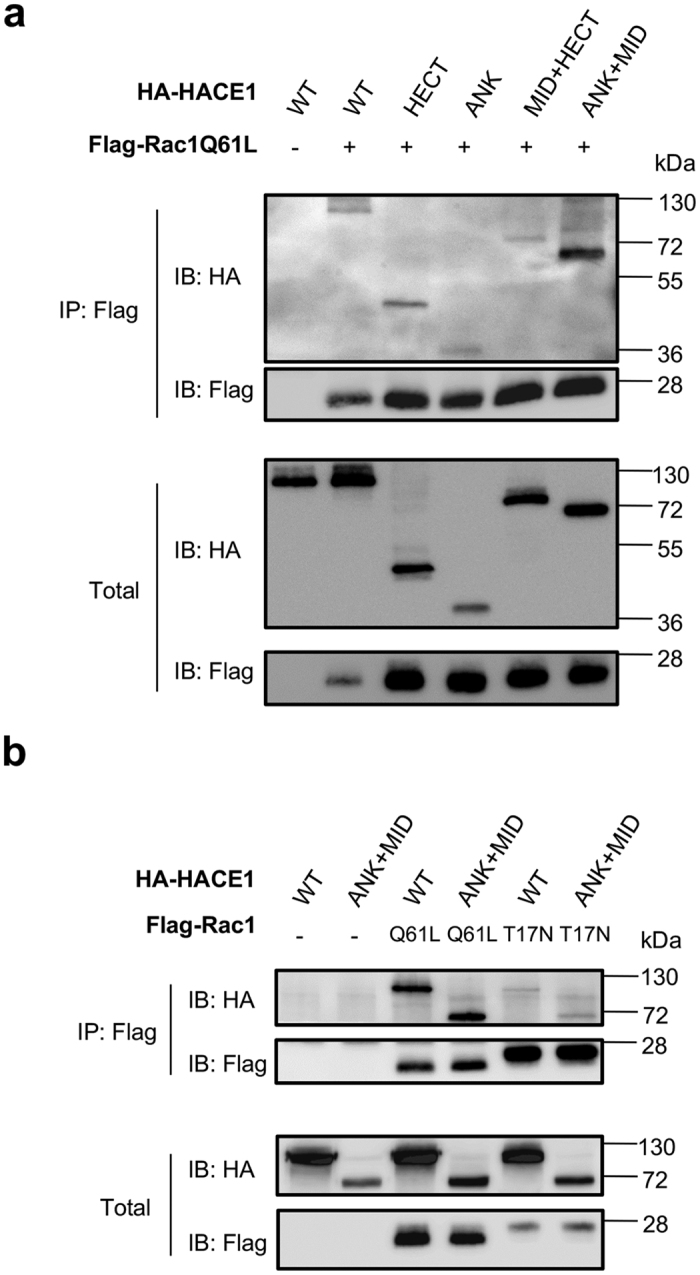
Binding of ANK + MID domain to the activated form of Rac1. (**a**) Immunoprecipitations anti-Flag (IP: Flag) from cells co-transfected with expression vectors for Flag-Rac1 Q61L and various HA-tagged HACE1 constructs followed by immunoblots anti-HA (IB: HA) show the association of HACE1 domains with active Rac1. Immunoblots anti-Flag (IB: Flag) show levels of immunoprecipitated Rac1 Q61L. Immunoblots on total lysates show expression of constructs. (**b**) Co-immunoprecipitations performed between either Flag-Rac1 Q61L (active) or Flag-Rac1 T17N (inactive) and HA-tagged HACE1wild-type (WT) or ANK + MID domains.

Our analysis of cancer-associated mutations in HACE1 pointed to a critical role of the ANK domain in coordinating the association of HACE1 with Rac1 for its ubiquitylation by the HECT domain. The position of the MID domain between the ANK and HECT domains raises the possibility that it may contribute to the recognition of Rac1. To test this hypothesis, we performed a series of co-immunoprecipitations between the active form of Rac1 and the different HACE1 domains. This revealed that active Rac1 binds to both the ANK and HECT domains of HACE1 (Fig. 6a). Although the MID domain of HACE1 alone did not interact with active Rac1 (not shown), we found that it modulates the capacity of ANK and HECT domains to interact with Rac1. Indeed, we measured that MID + HECT had a lower capacity to bind to active Rac1 compared to the HECT domain alone, whereas ANK + MID showed an increased association compared to the ANK domain alone (Fig. 6a). We further analyzed the specificity of binding of ANK + MID to the active form of Rac1. We observed that the ANK + MID domains associated specifically with active Rac1, whereas the ANK, HECT and MID + HECT domains were less sensitive to the activation status (Fig. 6b and Supplementary Figure S5). These experiments unravel the cooperation between the ANK and MID domains for binding to Rac1, and establish a molecular function of the MID domain in restricting the specificity of association of HACE1 to active Rac1.

## Discussion

Our work uncovers several missense mutations in *hace1* ANK and MID domains, identified in cancer samples, that diminished its capacity to control cell proliferation. Furthermore, structural modeling allowed us to define a surface epitope in the vicinity of Gly-175, which controls the association of HACE1 with Rac1 for ubiquitylation. In addition, we identify an ancillary role for the MID domain in defining the specificity of HACE1 for the active form of Rac1. Collectively, our data identify amino acids outside the catalytic HECT domain that are essential for the control of cell proliferation by HACE1, some of which are critical for the regulation of Rac1 by ubiquitylation.

Previous studies have implicated the E3 ubiquitin ligase activity of HACE1 in the control of cell growth and the regulation of Rac1^5^,^10^,^13^. We reasoned that some of the mutations in cancer samples targeting *hace1* should bear genetic traces of a rupture of the functional relationship between the tumor-suppressive function of HACE1 and the cell growth-promoting activity of Rac1. Notably, whereas a clear genetic link has been established between the modulation of expression of several HECT domain-containing ubiquitin ligases and cancer, little is known on the presence of pejorative mutations of this type of ubiquitin ligase in cancer^3^. The catalogue of somatic mutations in cancer is designed to store and display somatic mutation information relating to human cancers^17^. Here, we analyzed a series of 13 cancer-associated somatic missense mutations in ANK + MID domains (1–545) that were reported in the datasets of COSMIC version 64. The current version 79 of the database now displays 55 missense mutants in this part of HACE1. We note that mutations G175C and A204T no longer appear in this version of COSMIC, but they are now listed in the cBioPortal site for Cancer Genomics hence remain valid for our analysis (http://www.cbioportal.org)^18^,^19^. By analyzing the COSMIC data sets, we noticed an absence of hot spot of mutations in *hace1*. Indeed, only R332Q appeared with an occurrence of three mutants in the latest version 79 of COSMIC. Nevertheless, we noticed that 50% of all mutants in the ANK domain (9 out of 18, COSMIC version 79) localized in the repeats 5 and 6 (Supplementary Figure S6). The focal distribution of mutants in ANK 5 and 6 together with the present study suggest that this region is of critical importance in the mechanism that link HACE1 ubiquitylation of Rac1 to its control of cell proliferation. Up-dated versions of COSMIC and cbioportal now report mutations of HACE1 associated with breast cancer notably at position Gln-110, in the vicinity of the position Lys-111 we have pinpointed in our study. Remarkably, this version of COSMIC also reports a new position mutated in cancer, Gln-173, which corresponds to a position that we have identified in our 3D modeling and functional analysis, as being essential in the control of Rac1 ubiquitylation.

The 13 cancer-associated missense substitutions reported in the COSMIC cancer library version 64 that we analyzed are located along the ANK and MID domains. Mutations with an impact on the binding and ubiquitylation of Rac1 were identified in the ANK domain only. While our interaction mapping approach clearly demonstrated the involvement of the MID domain for the selectivity and efficiency of binding of HACE1 to the active form of Rac1, none of the mutations in the MID domain impacted HACE1 ubiquitin ligase activity on the GTPase. It is interesting to note that HACE1 ubiquitylates Rac1 on Lys147^20^, which is remote from the regions that are structurally sensitive to the bound nucleotide; furthermore, this residue is fully accessible in inactive Rac1 bound to RhoGDI^21^. In the absence of elements in HACE1 that recognize the protein is in its active, GTP-bound form, this would result in unregulated Rac1 ubiquitylation and degradation, which would be detrimental given the essential function of Rac1. Thus, additional structural elements are needed for HACE1 to recognize Rac1 in its active state. Our data favor a model in which the ANK domain binds Rac1 without discriminating between the guanine-nucleotide bound forms, and that specificity is provided by the MID domain. We note that since the MID domain is unrelated to any domain of known structure hence cannot be modelled reliably, we cannot rule out that mutations in this domain have a direct or indirect effect on HACE1 folding. In contrast, the ankyrin repeats can be reliably modelled, which allowed us to rule out that the effect of mutations is due to unfolding effects in this region. Accordingly, we could use the 3D model to define exposed residues in the vicinity of Gly-175 that form a surface epitope and show that this epitope controls HACE1 activity on Rac1. Furthermore, we found that substitutions with different amino acids at the same location (ie Val-140) can result in increased or decreased HACE1 activity on Rac1 depending on the size of the substituted side chain, confirming that these mutations do not impair the structural integrity of HACE1 and underscoring the fine and critical role of the mutated residue in HACE1 function.

In conclusion, we here reveal the presence in *hace1* of missense mutations in cancer samples that alter its capacity to control cell proliferation and a cluster of amino-acids in ankyrin repeats 5 to 7 that controls Rac1 binding and ubiquitylation.

## Methods

### Plasmid constructs

Human pXJ-HA HACE1 WT and C876S expression plasmids are described in ref. 11. cDNAs were subcloned into pKH3-HA3 and pRK5-myc plasmids by using *Bam*HI/*Eco*RI restriction sites. HACE1 mutants were obtained by mutagenesis (QuickChange Lighting, Agilent) using primers listed in the supplementary Table 2. pXJ-HA-Rac1 Q61L and pRK5-myc-Rac1Q61L plasmids are described in refs 15 and 22. For infection experiments we used the pMSCV-HA-HACE1 WT retroviral vector^5^, in which we generated HACE1 point mutations (QuickChange Lighting, Agilent). All constructs were verified by DNA sequencing.

### Cell culture, growth measurement, infection and transfection

Chinese hamster epithelial ovary cells (CHO-K1, CCL-61, ATCC) were grown and transfected as described in refs 23 and 24. MCF-12A mammary gland epithelial cells (CRL-10782, ATCC) were maintained in DMEM/F12 supplemented with 5% horse serum (Biowest), recombinant human Epidermal growth factor (20 ng/mL) (Peprotech), human recombinant insulin (10 μg/mL) (Life Technologies), hydrocortisone (0.5 μg/mL) (Sigma-Aldrich), and cholera toxin (100 ng/mL) (Sigma-Aldrich). MCF7 mammary gland adenocarcinoma (ATCC, HTB-22) were cultured in DMEM with 10% fetal calf serum and human recombinant insulin (10 μg/mL) (Life Technologies). Cell lines were transduced as described in ref. 25. Briefly, pMSCV retroviral vector constructs were transfected into the packaging cell line Phoenix-Ampho (ATCC, CRL3213) using Lipofectamine 2000 (Invitrogen). Viral supernatants (3 ml from 60 mm dish) were harvested after 36 hours and added to MCF12A or MCF7 cells grown in T25 flasks. After 24 hours the virus-containing medium was replaced by culture medium and after another 24 hours (MCF-12A) or 72 hours (MCF7) the selection of stably transduced cells was initiated by adding Hygromycin B (SIGMA-Aldrich) at 0.05 mg/ml for MCF-12A and 0.1 mg/ml for MCF7. Cell proliferation was assessed using a Colorimetric Cell Viability Kit (CCVK-1, PromoCell) on cells cultured between passages 2 to 4. Cells were seeded in 96-well culture plates at 3 000 cells for MCF-12A and 4 000 MCF7 cells per well and incubated at 37 °C with medium replacement every two days. Cell proliferation was assessed by the ability of metabolically active cells to reduce tetrazolium salt (WST-8) to yellow-colored formazan. Cells were exposed to WST-8 for 1 h and the absorbance was measured (wavelength 450 nm). We assessed colony formation in 96-well plates with 5000 transduced cells/well using the CytoSelect Cell Transformation assay (Cell Biolabs). Colonies were allowed to form for 10 days.

### Immunoprecipitation and His_6_-tagged ubiquitin precipitation procedures

For *in vivo* ubiquitylation measurements, CHO cells (5 × 10^6^) were transfected with 5 μg of His-tagged ubiquitin expression vector together with 5 μg of HA-tagged Rac1 Q61L and either myc- tagged HACE1 WT or mutant constructs. Ubiquitylated proteins were purified by His-tag affinity purification on cobalt resin in denaturing conditions, as described in ref. 24. For immunoprecipitation experiments, HEK293T cells (3 × 10^6^) were transfected using 5 μg of pRK5-myc-Rac1 Q61L and 5 μg of pKH3-HA3-HACE1 WT or mutants, as indicated in the figure legends. Cells were lysed in 1 ml of immunoprecipitation buffer (IPB) (25 mM Tris-BASE pH 7.5, 150 mM NaCl, 5 mM MgCl_2_, 0,5% (v/v) Triton-X100, 10 mM NaF, 1 mM phenylmethylsulfonyl fluoride, 2 mM Na_3_VO_4_, 2 mM DTT and 4% (v/v) glycerol). Cleared lysates were incubated with 5 μg of mouse anti-c-myc antibody for 3 hours at 4 °C. Immunoprecipitates were immobilized on Dynabeads^®^ Protein G (Invitrogen) for 30 min at 4 °C. Beads were washed three times with 1 ml of IPB and resuspended in 50 μl of Laemmli buffer. Proteins were resolved on 12% SDS-PAGE. Primary antibodies used were mouse anti-HA (HA.11 clone 16B12 monoclonal antibody, Covance), anti-c-myc (clone 9E10, Roche), anti-Rac1 (clone 102, BD Transduction laboratories) and anti-Flag (clone M2, SIGMA) or rabbit polyclonal anti-GAPDH (Santa-Cruz). Secondary antibodies used were Goat HRP-conjugated anti-mouse or swine HRP-conjugated anti-rabbit secondary antibodies (DAKO). Signals were imaged and quantified using the Fujifilm LAS-3000 system and Multi Gauge V3.0 software.

### Structure Modeling

The model of HACE1 was calculated with the protein structure prediction software Rosetta as implemented in the Robetta Server (http://robetta.bakerlab.org/)^26^ and analyzed with the PyMOL molecular graphics system^27^. The ankyrin repeat module was arbitrarily defined such that the first repeat starts at residue 1.

### Statistical Analysis

Data were analyzed with Prism 5.0b (GraphPad Software) by one-way ANOVA with Dunnett’s multiple comparison test and unpaired student t-test (*p < 0.05; **p < 0.01, ***p < 0.001, ****p < 0.0001).

## Additional Information

**How to cite this article**: Andrio, E. *et al*. Identification of cancer-associated missense mutations in *hace1* that impair cell growth control and Rac1 ubiquitylation. *Sci. Rep.* **7**, 44779; doi: 10.1038/srep44779 (2017).

**Publisher's note:** Springer Nature remains neutral with regard to jurisdictional claims in published maps and institutional affiliations.

## Supplementary Material

Supplementary Information
