# Supplementary material for: Identification of cancer-associated missense mutations in hace1 that impair cell growth control and Rac1 ubiquitylation
Source: Sci Rep. 2017 Mar 20;7:44779. doi: 10.1038/srep44779 (PMC5357957; doi:10.1038/srep44779)
Supplement: Supplementary Information [file srep44779-s1.pdf]

Identification of cancer-associated missense mutations in *hace1* that impair cell growth control and Rac1 ubiquitylation

Author list

Emilie Andrio, Romain Lotte, Daniel Hamaoui, Jacqueline Cherfils, Anne Doye, Mads Daugaard, Poul H. Sorensen, Frédéric Bost, Raymond Ruimy, Amel Mettouchi and Emmanuel Lemichez

Supplementary information

Supplementary Figure S1: Control of HA-HACE1 mutant expression

(**a**) Expression of HA-tagged HACE1 WT and mutants in MCF-12A cell lines was controlled by immunoblots anti-HA (IB: HA). (**b**) Impact of expression of HA-HACE1 WT and M114V, G175S, G175C and A204T mutants on Rac protein level in MCF-12A cell lines. Proteins were detected by immunoblotting anti-HA and anti-Rac. Graph shows quantification of Rac signals normalized to GAPDH and expressed as fold changes to vector. (**c**) Expression of HA-tagged HACE1 WT and mutants in MCF7 cell lines was controlled by immunoblots anti-HA. (**a-c**) Immunoblot anti-GAPDH on total cell lysates was performed as loading control.

Supplementary Figure S2: Efficiency of Rac1 ubiquitylation by HACE1 G175N

Immunoblots showing levels of ubiquitylation of Rac1 Q61L catalyzed by HACE1 WT, G175S and G175N mutants. Precipitated His_6-tagged ubiquitylated proteins (HisP) were immunoblotted anti-HA to reveal Rac1 Q61L ubiquitylation profiles. Immunoblots anti-HA and anti-myc on total cell lysates show protein expression and anti-actin is used as loading control. Each value corresponds to mean of three independent measurements analyzed by unpaired t-test with *$p \leq 0.05$ or non-significant (ns).

Supplementary Figure S3: Ability of HACE1 V140A to control cell proliferation

(**a**) Graphs show kinetics of cell proliferation. MCF-12A cell lines expressing HA-HACE1 wild-type or V140A. Each value corresponds to mean values of two independent experiments with technical quadruplicate analyzed by one-way ANOVA Dunnett's multiple comparison test with ***$p \leq 0.0003$, *$p \leq 0.015$ or non-significant (ns). (**b**) Immunoblot anti-HA show HACE1 WT and V140A expression.

Supplementary Figure S4: Rac1 association with HACE1 mutants

Association of myc-Rac1 Q61L with HA-tagged HACE1 wild-type (WT) and mutants was assessed by immunoprecipitation anti-myc (IP: myc) followed by immunoblots anti-HA (IB: HA) in HEK293 cells. Immunoblot anti-myc (IB: myc) shows levels of immunoprecipitated Rac1. Immunoblots on total lysates (Total) show expression of constructs.

Supplementary Figure S5: Binding of HACE1 domains to active Rac1

Association of HACE1 domains described in the panel with either Flag-Rac1 Q61L (active) or Flag-Rac1 T17N (inactive) assessed by immunoprecipitation anti-Flag (IP: Flag) followed by immunoblots anti-HA (IB: HA). Cells were co-transfected with expression vectors of Flag-Rac1 Q61L or T17N and various HA-tagged HACE1 constructs. Immunoblots anti-Flag (IB: Flag) show levels of immunoprecipitated Rac1. Immunoblots on total lysate show expression of constructs.

Supplementary Figure S6: Cancer-associated mutations in ANK domain

Histogram of repartition of cancer-associated missense mutations in each ankyrin repeat 1 to 7. Analysis conducted on data sets from the COSMIC version 79.

Supplementary Table 1: Cancer-associated somatic mutations in HACE1 1-545

Mutation (Amino acid)	Mutation (DNA)	Domain of HACE1	Primary tissue/ Histopathological type of cancer	References
**K111N**	G333T	ANK	Large intestine/carcinoma	[19]
**M114V**	A340G	ANK	Ovary/carcinoma	[19,28]
**A162V**	C485T	ANK	Ovary/carcinoma	[19,28]
**G175C**	G523T	ANK	Lung/carcinoma	[19]
**G175S**	G523A	ANK	Skin/carcinoma	[19,29]
**A204T**	G610A	ANK	Large intestine/carcinoma	[19]
**Y251H**	T751C	ANK	Large intestine/carcinoma	[19]
**H252N**	C754A	ANK	Ovary/carcinoma	[19,28]
**T263A**	A787G	MID	Large intestine/carcinoma	[19,30]
**R332Q**	G995A	MID	Large intestine/carcinoma	[19,31]
**R353G**	A1057G	MID	Large intestine/carcinoma	[19,30]
**P359S**	C1075T	MID	Pancreas/carcinoma	[19,32]
**R493I**	G1478T	MID	Large intestine/carcinoma	[19,30]

Supplementary Table 2: Sequence of the primers used to generate HACE1 mutants

Mutation<br>Amino acid	Mutation<br>DNA	Forward primers<br>(5'-3' sequence)	Reverse primers<br>(5'-3' sequence)
**HACE K111N**	G333T	tggcagcaagaaatgggcaga ataaatgtatgagtaaattattaga	tctaataatttactcatacatttattctgcc catttcttgctgcca
**HACE1 M114V**	A340G	ctaataatttactcacacatttcttct gcccatttcttgctgcc	ggcagcaagaaatgggcagaagaa atgtgtgagtaaattattag
**HACE1 V140A**	T419C	acattggctggctgcgaatgggc ggacag	catgttgcctgccagaacaatcacaa gacgacagtgca
**HACE1 V140L**	G418T	acagcaatacattggctggctttg aatgggcgga	tccgcccattcaaagccagccaatgt attgctgt
**HACE1 A162V**	C485T	gttgatgttgaggatgtcatgggg cagacagca	tgctgtctgccccatgacatcctcaaca tcaac
**HACE1 Q173A**	C517G, A518C	cgtcttgtgaccgttcgcgcaggc aacatgcag	tgcatgttgcctgcgcgaacggtcaca agacg
**HACE1 N174A**	A520G, A521C	tgtcgtcttgtgaccggcctggca ggcaacatgc	gcatgttgcctgccaggccggtcaca agacgaca
**HACE1 G175C**	G523T	gttgcctgccagaactgtcacaa gacgacag	ctgtcgtcttgtgacagttctggcaggc aac
**HACE1 G175N**	G522A, G523A	tgcactgtcgtcttgtgattgttctgg caggcaacatg	catgttgcctgccagaacaatcacaa gacgacagtgca
**HACE1 G175S**	G523A	ctgtcgtcttgtgactgttctggcag gcaac	gttgcctgccagaacagtcacaagac gacag
**HACE1 A204T**	G610A	caggagcaactccattgtacttta cttgcagtcatgg	ccatgactgcaagtaaagtacaatgg agttgctcctg
**HACE1 Y251H**	T751C	aagcctcgggtgatgttgaattaat acttcacaagtctctc	gagagacttgtgaagtattaattcaac atcacccgaggctt
**HACE1 H252N**	C754A	gaaaaagcctcgggttatattgaa ttaatacttcacaagtctctc	gagagacttgtgaagtattaattcaata taacccgaggctttttc
**HACE1 T263A**	G787A	tttcagactattattcaaatggcac agaatgaagacctccgag	ctcggaggtcttcattctgtgccatttga ataatagtctgaaa
**HACE1 R332Q**	G995A	gggaggatggaccaatttgaaa gacgtgacaaaac	gttttgtcacgtctttcaaattggtccatc ctccc
**HACE1 R353G**	A1057G	aatgggaataaaactccaggaa gccaggtgttcaagc	gcttgaacacctggcttcctggagtttta ttcccatt
**HACE1 P359S**	C1075T	gtgccaaagcaattccagactctt gaacacctggcttctt	aagaagccaggtgttcaagagtctgg aattgctttggcac
**HACE1 R493I**	G1478T	tctgcaaacatgatgaagttttaaa atgctttgttaatataaatcccaaa attatatttgac	gtcaaatataattttgggatttatattaac aaagcattttaaaacttcatcatgtttgc aga

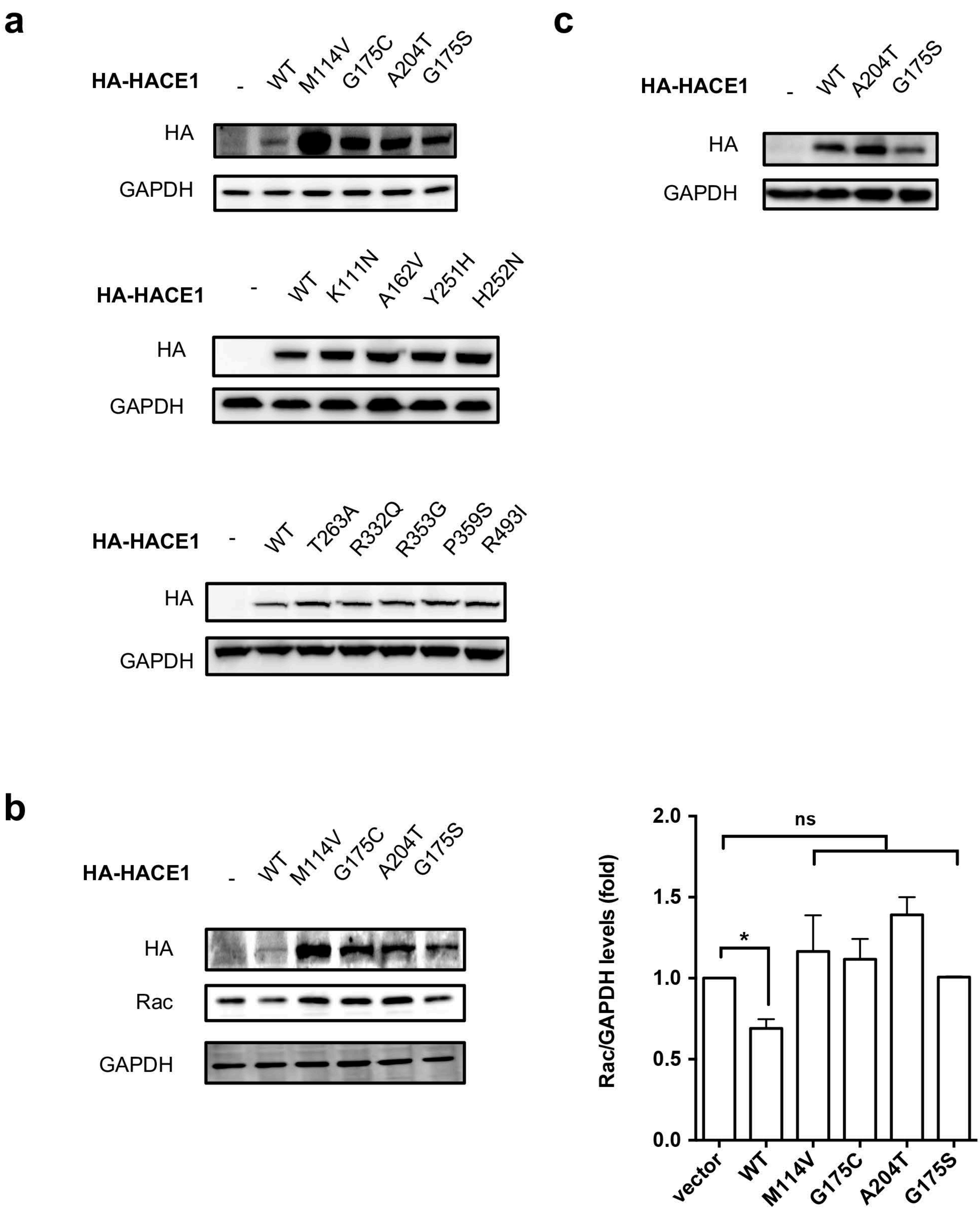
a
HA-HACE1
-
WT
M114V
G175C
A204T
G175S
HA
GAPDH
HA-HACE1
-
WT
K111N
A162V
Y251H
H252N
HA
GAPDH
HA-HACE1
-
WT
T263A
R332Q
R353G
P359S
R493I
HA
GAPDH
b
HA-HACE1
-
WT
M114V
G175C
A204T
G175S
HA
Rac
GAPDH
Rac/GAPDH levels (fold)
2.0
1.5
1.0
0.5
0.0
ns
*
vector
WT
M114V
G175C
A204T
G175S
c
HA-HACE1
-
WT
A204T
G175S
HA
GAPDH

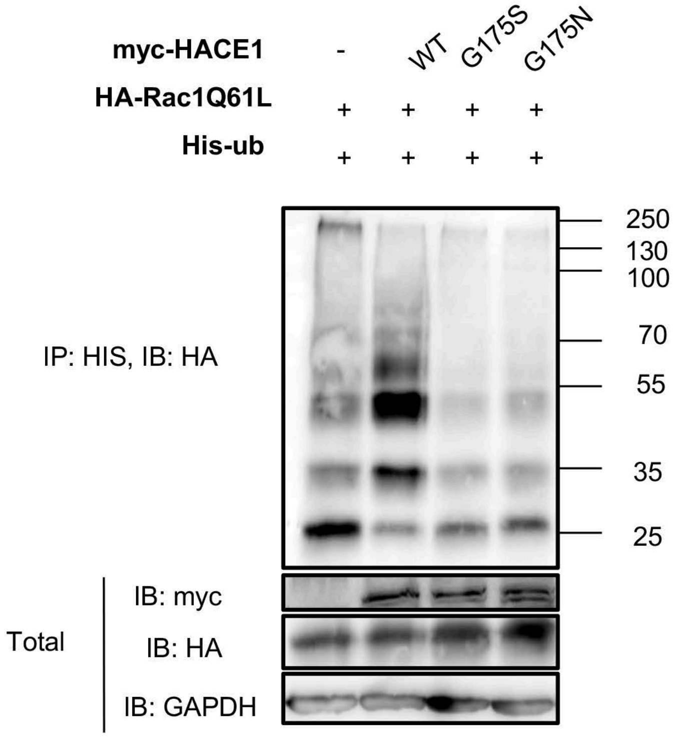
myc-HACE1
-
WT
G175S
G175N
HA-Rac1Q61L
+ + + +
His-ub
+ + + +
IP: HIS, IB: HA
250
130
100
70
55
35
25
Total
IB: myc
IB: HA
IB: GAPDH

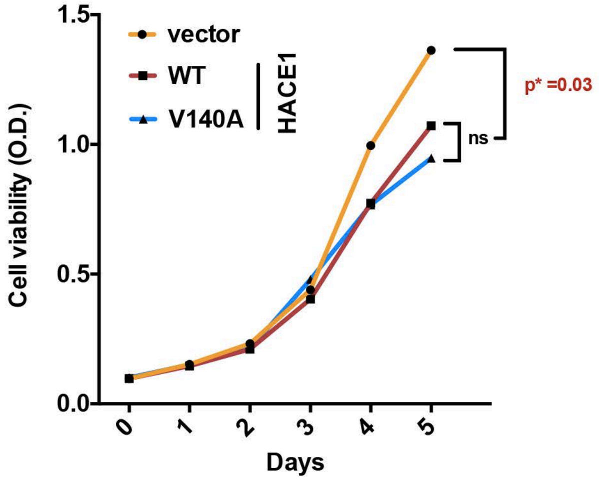
vector
WT
V140A
HACE1
p* =0.03
ns
Cell viability (O.D.)
1.5
1.0
0.5
0.0
0
1
2
3
4
5
Days

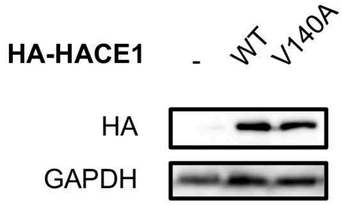
HA-HACE1
-
WT
V140A
HA
GAPDH

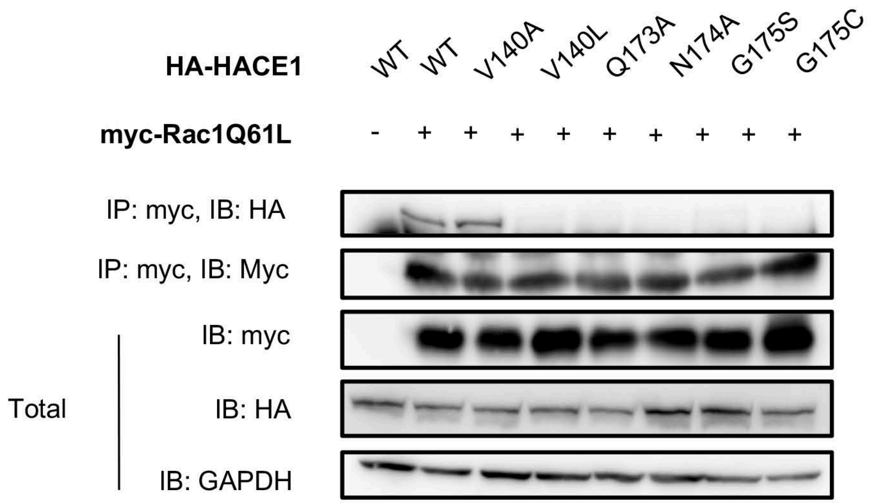
HA-HACE1
WT
WT
V140A
V140L
Q173A
N174A
G175S
G175C
myc-Rac1Q61L
- + + + + + + + + +
IP: myc, IB: HA
IP: myc, IB: Myc
IB: myc
Total
IB: HA
IB: GAPDH

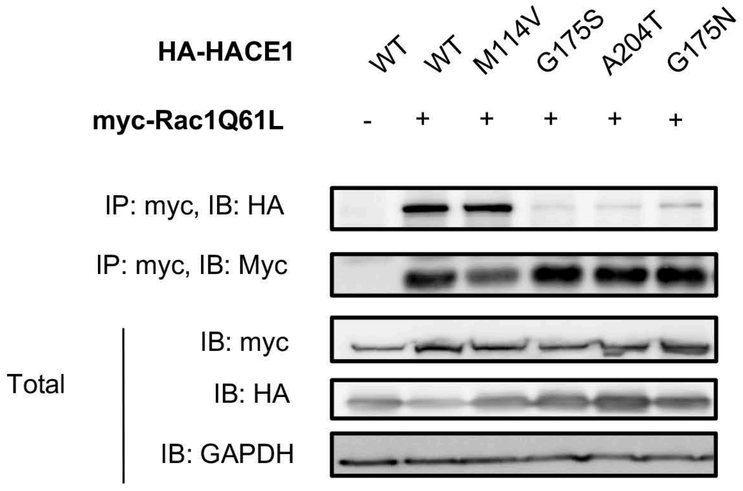
HA-HACE1
WT
WT
M114V
G175S
A204T
G175N
myc-Rac1Q61L
- + + + + +
IP: myc, IB: HA
IP: myc, IB: Myc
IB: myc
Total
IB: HA
IB: GAPDH

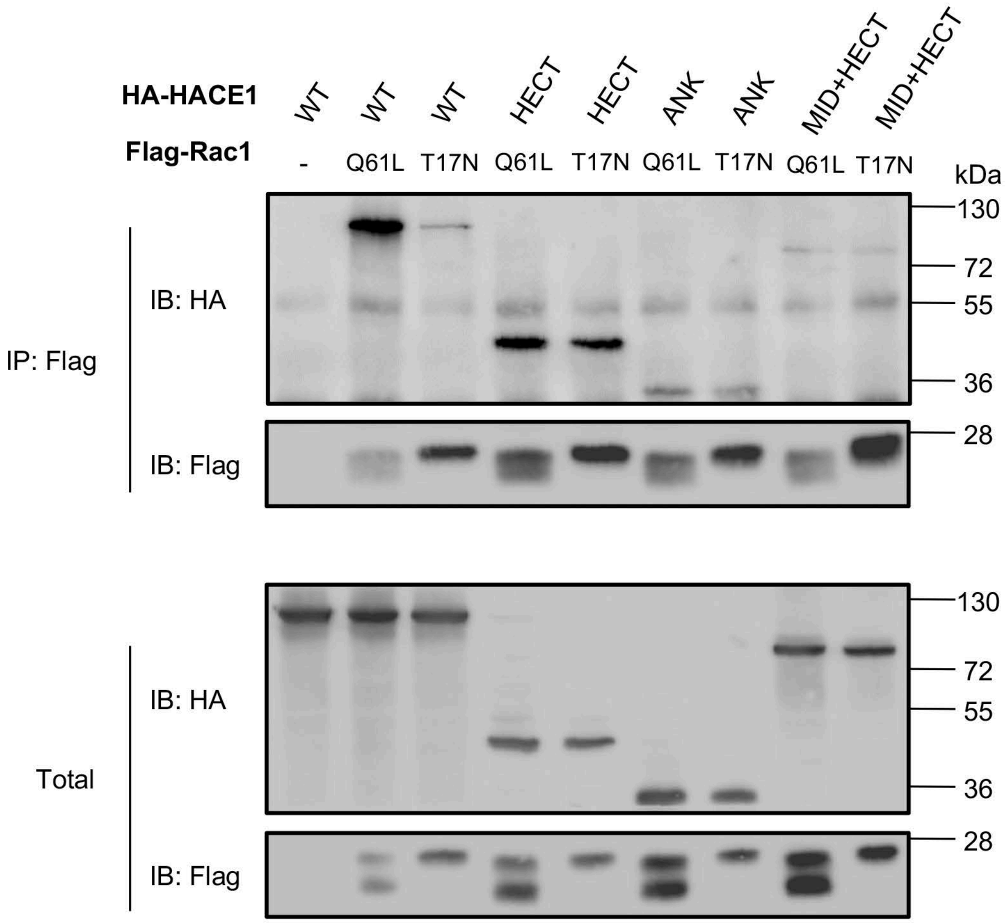
HA-HACE1
WT
WT
WT
HECT
HECT
ANK
ANK
MID+HECT
MID+HECT
Flag-Rac1
-
Q61L
T17N
Q61L
T17N
Q61L
T17N
Q61L
T17N
kDa
130
72
55
36
28
IB: HA
IB: Flag
IP: Flag
130
72
55
36
28
IB: HA
IB: Flag
Total

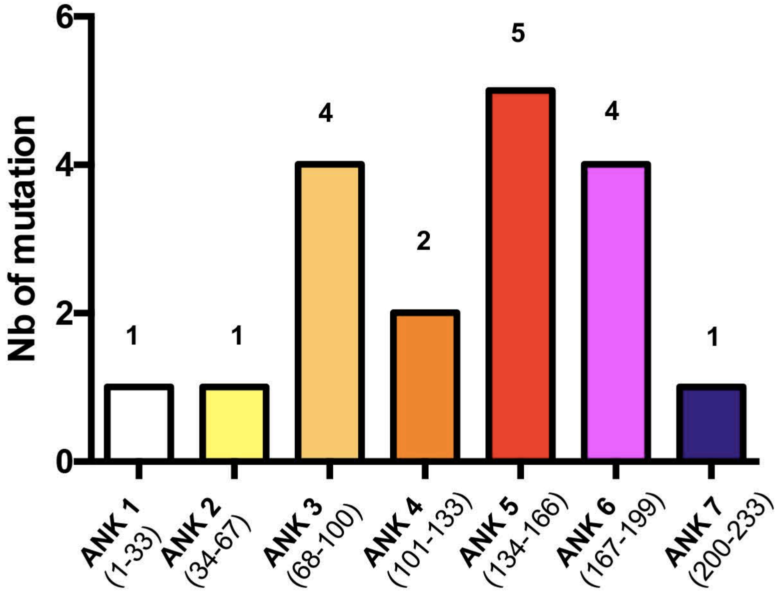
Nb of mutation
6
4
2
0
1
1
4
2
5
4
1
ANK 1 (1-33)
ANK 2 (34-67)
ANK 3 (68-100)
ANK 4 (101-133)
ANK 5 (134-166)
ANK 6 (167-199)
ANK 7 (200-233)
